# Mental and physical health burden and quality of life in Czech women with lipedema

**DOI:** 10.3389/fgwh.2025.1629077

**Published:** 2025-07-24

**Authors:** Monika Kunzová, Eliška Lagová, Leslyn Keith

**Affiliations:** ^1^ Department of Public Health, Faculty of Medicine, Masaryk University, Brno, Czechia; ^2^ International Clinical Research Center (ICRC), St Anne’s University Hospital (FNUSA), Brno, Czechia; ^3^ Lipedema Project, Inc., Boston, MA, United States

**Keywords:** lipedema, depression, quality of life, well-being, psychosocial burden

## Abstract

**Background:**

Lipedema is a chronic condition characterized by excessive fat deposition in the hips, buttocks, and lower legs, significantly impacting quality of life. In Czechia, limited research exists on the relationship between lipedema symptoms and depressive symptoms, despite the condition's prevalence and its impact on mental health.

**Aim:**

This study aims to investigate the relationship between lipedema symptoms and the severity of depressive symptoms among Czech women and assess their quality of life.

**Methods:**

We administered an online survey to 43 women with lipedema. Participants completed questionnaires on quality of life (WHOQOL-BREF), sociodemographic and clinical characteristics, and depressive symptoms severity, evaluated using the PHQ-9 tool.

**Results:**

PHQ-9 results showed that 50.9% of participants exhibited moderate to severe depressive symptoms. Quality of life ratings varied, with 27.9% of participants rating their health as poor or very poor. Significant correlations were found between lipedema symptoms, such as shortness of breath, muscle stiffness, and depression severity, indicating a complex relationship between physical symptoms and mental health.

**Conclusion:**

These findings highlight the significant mental health burden faced by individuals with lipedema. The association between physical and depressive symptoms emphasizes the need for comprehensive, tailored interventions, especially integrated mental and physical healthcare approaches, aimed at improving overall well-being in this population.

## Introduction

Lipedema is a chronic condition predominantly affecting women, marked by abnormal fat accumulation in the hips, buttocks, and legs while sparing the feet. First identified in 1940 by Allen and Hines (1), lipedema is often underdiagnosed and commonly mistaken for obesity or lymphedema (2). Current estimates suggest that approximately 11% of women worldwide may be affected by this condition (3, 4), although rare cases are reported in men due to hormonal imbalances (5). Despite the condition's prevalence, understanding of lipedema's pathophysiology, causes, and treatment options remains limited (1–3, 5).

The exact cause of lipedema remains unclear (1–3, 5); however, it is thought to be influenced by genetic predisposition and hormonal factors, particularly during puberty, pregnancy, or menopause (2, 6–8). Inflammation, likely driven by abnormal estrogen receptor expression, leads to fibrosis, chronic pain, and swelling (1, 9, 10).

Lipedema is characterized by disproportionate fat accumulation in the lower limbs, sparing the feet, often with symmetrical distribution. The condition typically emerges during hormonal changes such as puberty, pregnancy, or menopause. Affected areas are tender and painful upon pressure, bruise easily due to capillary fragility, and show minimal or no response to weight loss interventions, distinguishing lipedema from general obesity. The absence of pitting edema and a negative Stemmer's sign are key differentiators from lymphedema (11–13).

Beyond the physical burden, lipedema is associated with significant psychological distress. Studies suggest that individuals with lipedema have higher rates of depressive symptoms, anxiety, and reduced quality of life compared to the general population (3, 14). Patients with more advanced lipedema stages report more physical and mental health issues, as well as negative experiences with healthcare providers (15). The chronic pain, mobility limitations, and lack of effective conservative treatment options contribute to feelings of helplessness and social withdrawal. These symptoms not only affect mobility and daily functioning but also contribute to a substantial psychological burden, with many patients experiencing low self-esteem and social isolation (1, 9, 10). In fact, a survey of 120 women with lipedema found that 94% experienced daily pain, with 66% reporting pain as moderate to severe (10).

Additionally, many patients face stigmatization and misdiagnosis, often being incorrectly labeled as obese and advised to lose weight, despite the condition being unresponsive to conventional weight-loss strategies. This can lead to negative interactions with healthcare providers and a reluctance to seek medical care, further exacerbating mental health struggles (3, 16).

Given its diagnostic challenges, treatment options for lipedema remain limited (12, 17). Non-invasive approaches such as complete decongestive therapy, lymphatic drainage, compression therapy, and lifestyle changes are common, though invasive surgical treatments are increasingly sought to manage pain. However, these approaches do not cure the disease, and they carry significant risks of complications (3, 11, 18).

In Czechia, the understanding of how lipedema symptoms relate to mental health, particularly depressive symptoms, is limited. Despite the potential psychological burden, no study has explored this connection within the Czech population. A recent study conducted in Poland (14) has highlighted this connection, and given the shared cultural context, comparing these findings could reveal critical regional variations. This study aims to fill this gap by examining the relationship between lipedema symptoms and depressive symptoms severity among Czech women, contributing to the broader understanding of lipedema's physical and mental health implications and guiding potential healthcare interventions.

## Methods

From February to April 2024, an online survey was conducted targeting women in Czechia diagnosed with or showing symptoms of lipedema. Due to the absence of a national registry for lipedema in Czechia, the sample size was determined based on feasibility rather than formal power calculations. In light of the exploratory nature of this study, we aimed to recruit as many participants as possible within the data collection period. Future research should strive for larger, more representative samples accompanied by formal sample size estimations. Participants were recruited from a Czech Facebook group dedicated to individuals with lipedema, with approximately 900 members at the time of the study. The total number of people with lipedema in Czechia is unknown due to the absence of official prevalence data, but the estimated global prevalence is roughly 11% among women. As such, the study sample likely represents only a small fraction of those affected in the country. While recruitment through social media may introduce some bias, it remains a practical approach for engaging individuals with rare or underdiagnosed conditions, as demonstrated in prior studies (14).

Participation was voluntary and without financial incentives. Participants were eligible if they reported a physician-confirmed lipedema diagnosis or self-reported symptoms consistent with established criteria (19). Following the approach used in prior research (14), those with self-reported symptoms completed a structured questionnaire assessing hallmark features of lipedema (symmetrical fat distribution, pain, tenderness, easy bruising, and sparing of hands and feet). No clinical validation via medical records or physician confirmation was conducted, representing a limitation acknowledged in the Discussion.

Participants who did not complete the survey were excluded from the analysis. The survey was designed to capture comprehensive demographic, clinical, and quality-of-life information relevant to lipedema and depression among participants. It included the following sections:

### Demographic and clinical questionnaire

Participants provided information on age, lipedema onset (e.g., puberty, pregnancy), and comorbidities, offering insight into their demographic and clinical profiles. They were also asked to provide weight, height, waist, and hip circumference, which were used to calculate body mass index (BMI) and waist-to-hip ratio (WHR).

### Quality of life assessment

Quality of life was measured using the Czech version of the World Health Organization Quality of Life (WHOQOL-BREF) scale (20), which evaluates four domains: physical health, psychological health, social relationships, and environment. The Czech version of the WHOQOL-BREF has demonstrated good psychometric properties, with acceptable internal consistency across its four domains (21). The scale includes two additional items assessing overall quality of life and health, with higher scores indicating a better quality of life.

### PHQ-9 (patient health questionnaire-9)

The severity of depressive symptoms was evaluated using the Czech version of PHQ-9, a standardized tool that asks participants to report the frequency of depressive symptoms experienced over the past 28 days. Higher scores indicate greater symptom severity, with scores of 10 or above classified as moderate to severe depressive symptoms, consistent with mental health screening standards. The PHQ-9 has demonstrated satisfactory reliability and validity (22). The Czech version of the Patient Health Questionnaire-9 (PHQ-9) has been also validated as a reliable screening tool for depressive symptoms (23).

### Lipedema symptom severity

Participants reported the severity of 16 lipedema-related symptoms, including leg heaviness, swelling, bruising, and pain, on a 5-point Likert scale (0 = no problem, 4 = extreme severity). This symptom-focused data allowed a nuanced analysis of physical symptom severity and its association with depression. The questionnaire used to assess lipedema symptoms was adapted from previous studies (14, 15). While no formal validation of this specific version exists, similar symptom-focused assessments have been successfully used in prior lipedema research.

### Statistical analysis

The SPSS software (SPSS, version 28.0.0.0, IBM Corp.) was used for data analysis. Continuous variables were presented as mean and standard deviation. Differences between groups were assessed using chi-square test for categorical parameters. The level of statistical significance was set at *p* < 0.05

### Ethics

The principles laid by the Declaration of Helsinki for research involving human subjects and the General Data Protection Regulation (GDPR) of the European Union (EU) were followed while conducting the present study (24, 25). The study was exempted from the ethical review process, according to the opinion of the Ethics Committee, Faculty of Medicine, Masaryk University, as it was an entirely observational study with no anticipated harms. Informed consent was submitted electronically by all participants prior to their participation in this study as a prerequisite for displaying the self-administered questionnaire items. Identifying personal data were not collected from the participants to keep their identities anonymous. The participants were able to leave the study at any time without the need to justify their decision, and no responses were recorded until the participant finalized the entire self-administered questionnaire and confirmed to send out their responses.

Additionally, measures were taken to ensure the confidentiality and anonymity of the responses. We adhered to the principles of the Declaration of Helsinki and other relevant ethical guidelines to safeguard the well-being of participants.

## Results

**Figure 1:**
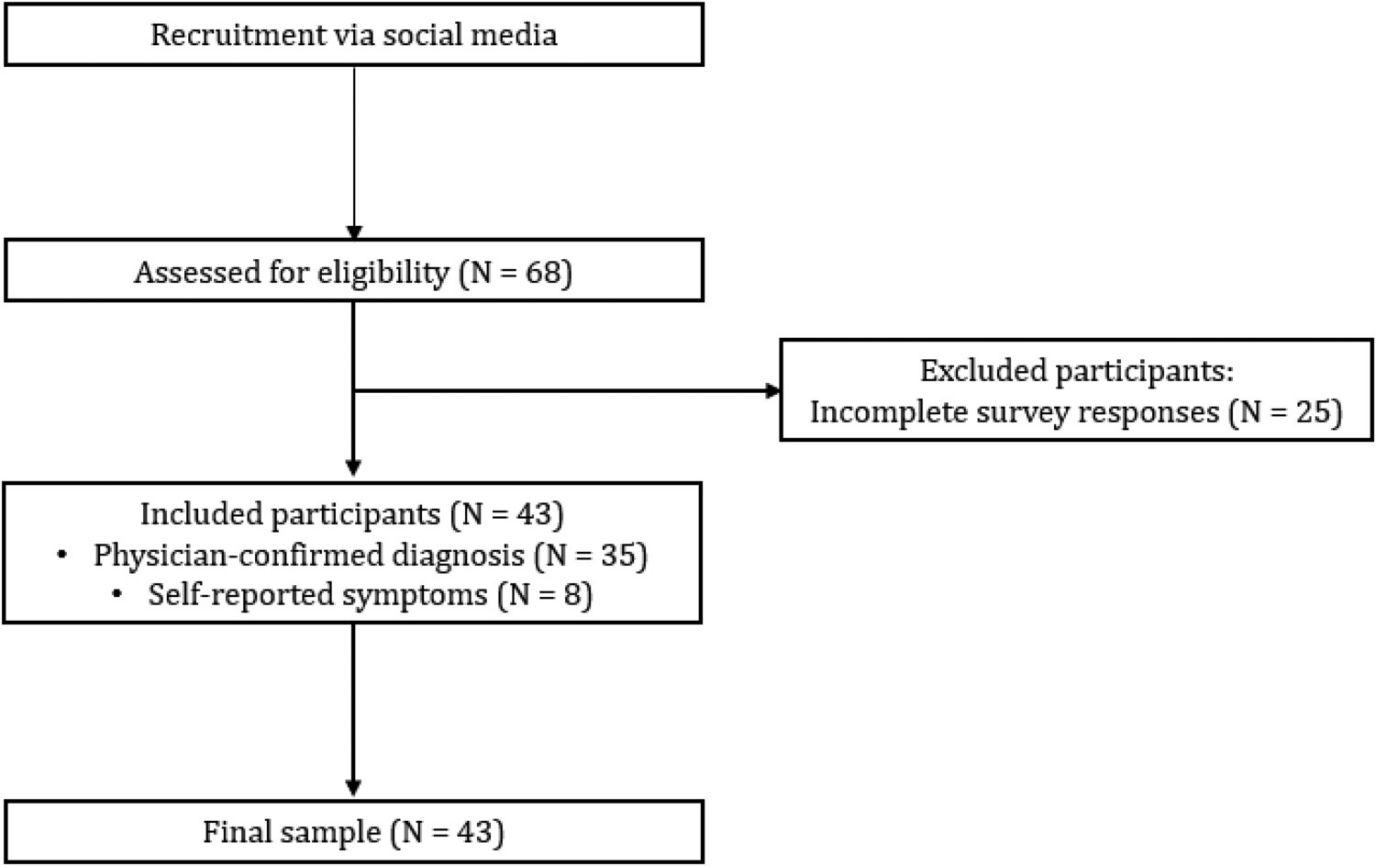
Workflow diagram of participant recruitment.

A total of 68 women initially participated in the study. Of these, 43 individuals who completed all required components of the survey were included in the final analytic sample (Figure 1). The participants ranged in age from 26 to 63 years, with a mean age of 42 (SD = 9.12). Among them, 81.4% (*N* = 35) reported receiving a formal diagnosis of lipedema. However, there were no significant differences in symptom severity between those formally diagnosed and those without a medical diagnosis. As a result, the analyses included the entire sample.

### Physical characteristics

The participants' mean Body Mass Index (BMI) was 33.9 (SD = 8.1), with 27.9% classified as overweight and 62.9% as obese. Waist-to-Hip Ratio (WHR) results showed that only 11.6% were classified as obese based on WHR, underscoring BMI's limitations in distinguishing lipedema from obesity (Table 1).

**Table 1:** Subject characteristics.

Age (y), mean (SD)	41.95 (9.12)
Confirmed diagnosis, N (%)	35 (81.4)
Initial source of information on lipedema, N (%)	
Online	24 (55.8)
Health professional	13 (30.2)
Other	6 (14.0)
Lipedema onset, N (%)	
Childhood	5 (11.6)
Puberty	15 (34.9)
Pregnancy	11 (25.6)
Menopause	2 (4.7)
Other	2 (4.7)
Hard to tell	8 (18.6)
Liposuction, N (%)	6 (14.0)
BMI, N (%)	
18.5-24.99 (normal weight)	4 (9.3)
25-29.00 (overweight)	12 (27.9
30-34.99 (obesity class I)	11 (25.6)
35-39.99 (obesity class II)	6 (14.0)
≥40 (obesity class III)	10 (23.3)
WHR, N (%)	
<0.85	38 (88.4)
≥0.85	5 (11.6)

BMI, body mass index; WHR, waist-to-hip ratio.

### Sources of information and lipedema onset

Most participants (55.8%) first learned about lipedema from online platforms, while 30.2% were informed by health professionals. Lipedema symptoms most commonly began during puberty (34.9%) or pregnancy (25.6%), suggesting hormonal influences on disease onset (Table 1).

### Physical activity and comorbidities

A notable 58.1% of participants engaged in over three hours of physical activity per week, with walking being the most common activity (34.9%). Comorbidities included venous insufficiency (34.9%), lymphedema (11.6%), and hypertension (9.3%), highlighting the multifaceted health challenges associated with lipedema (Table 2).

**Table 2:** Level and type of physical activity and comorbidities in women with lipedema.

Variable	Value
Level of physical activity (weekly), N (%)	
More than 3 h	25 (58.1)
1 to 3 h	13 (30.2)
Less than 1 h	4 (9.3)
None	1 (2.4)
Type of physical activity, N (%)	
Walking	15 (34.9)
Cycling	8 (18.6)
Aerobic exercise	5 (11.6)
Gym, Strength	4 (9.3)
Running	0 (0.0)
Yoga, Pilates	4 (9.3)
Aquatic exercise, swimming	5 (11.6)
Other	1 (2.3)
None	1 (2.3)
Self-reported Comorbidities, N (%)	
Hypothyroidism	3 (7.0)
Lymphedema	5 (11.6)
Venous insufficiency	15 (34.9)
Arthritis	0 (0.0)
Insulin resistance	0 (0.0)
Hypertension	4 (9.3)
Polycystic ovary syndrome	0 (0.0)
Irritable bowel syndrome	0 (0.0)
Fibromyalgia	1 (2.3)
None	14 (34.9)

### Lipedema symptoms

**Figure 2:**
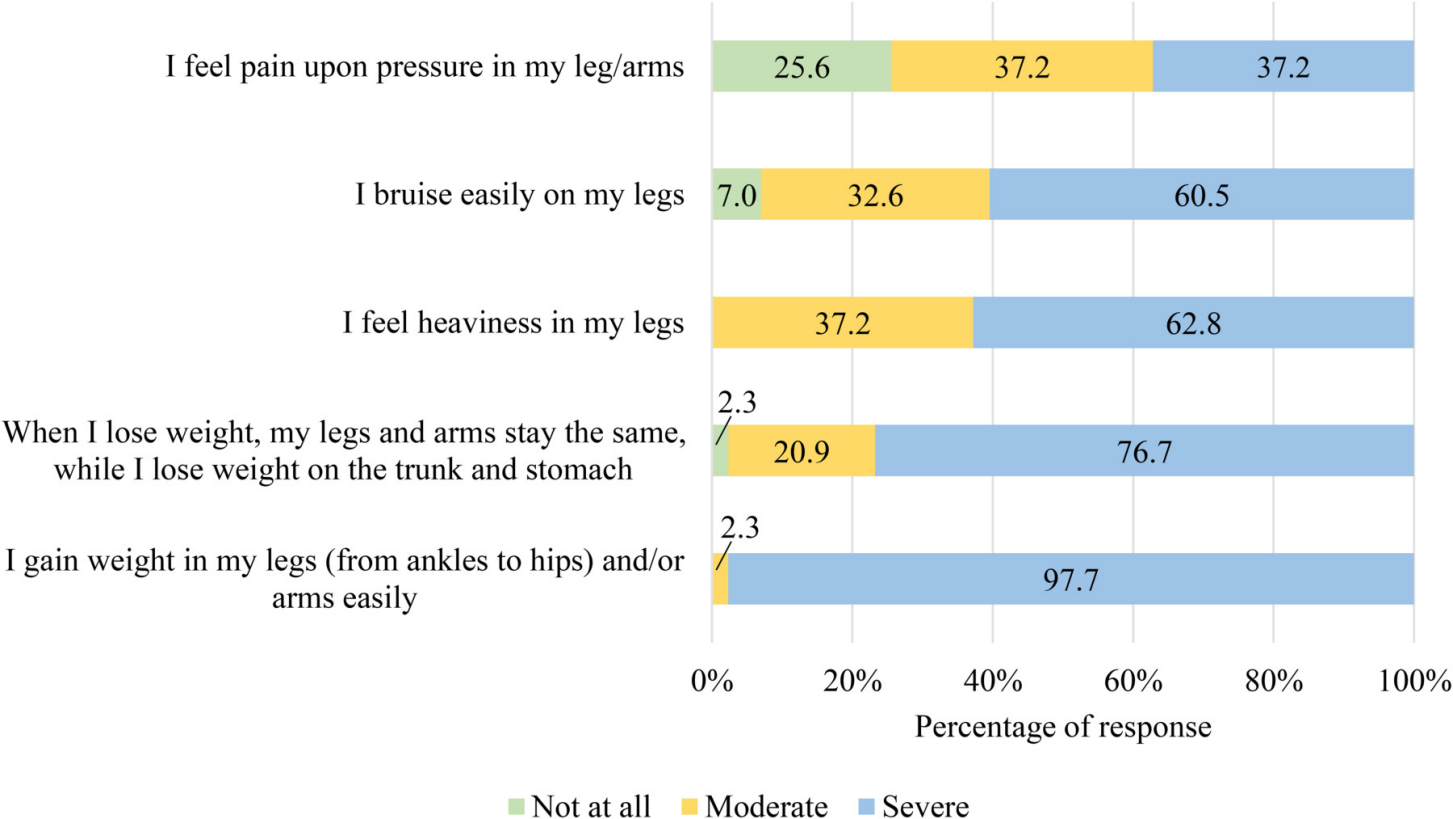
Participants’ ratings of intensity of symptoms based on lipedema symptoms criteria.

All participants (100%) reported gaining weight in their legs/arms easily (97.7% severe) and feeling heaviness in their legs (62.8% severe). In total, 97.6% found it difficult to lose weight from legs/arms (76.7% severe), 93.1% experienced easy bruising (60.5% severe), and 74.4% had pain upon pressure (37.2% severe) (Figure 2).

**Figure 3:**
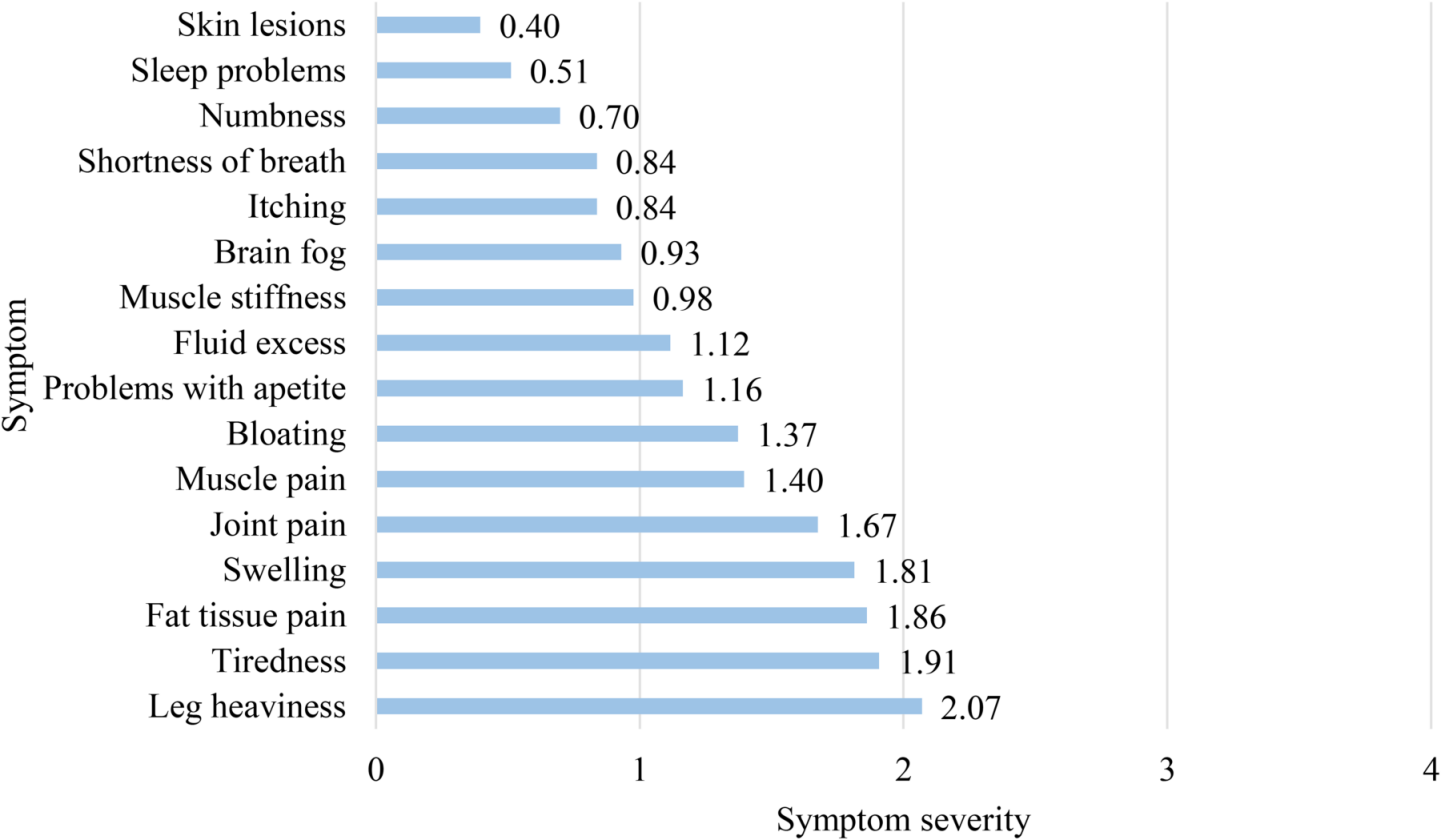
Participants’ mean scores for severity of lipedema symptoms.

Participants assessed 16 lipedema symptoms experienced over the past 28 days using a 5-point Likert scale, where 0 indicated no problem and 4 indicated extreme severity. The most severe symptoms included leg heaviness (M = 2.07, SD = 0.79), tiredness (M = 1.91, SD = 0.81), fat tissue pain (M = 1.86, SD = 0.91), and swelling (M = 1.81, SD = 0.90). A majority rated leg heaviness as moderate to extremely severe (72.1%), while over half reported moderate to severe tiredness (67.5%), fat tissue pain (65.1%), and swelling (63.5%) (Figure 3).

### Depression severity and quality of life

The PHQ-9 assessment revealed that 50.9% of participants had moderate to severe depressive symptoms, with 9.3% experiencing severe symptomatology (Table 3).

**Table 3:** Depressive symptom severity in 43 women with lipedema.

PHQ-9 score	n	%
≤4 (minimal depression)	10	23.3
5-9 (mild depression)	11	25.6
10-14 (moderate depression)	12	27.6
15-19 (moderately severe depression)	6	14.0
≥20 (severe depression)	4	9.3

Quality of life ratings, measured with the WHOQOL-BREF, were generally low: 39.5% rated their quality of life as moderate, while 30.2% rated it as poor or very poor. Health satisfaction was also low, with 30.2% expressing dissatisfaction and none indicating they were very satisfied (Table 4).

**Table 4:** Quality of life and satisfaction with health status.

Quality of life	n	%
Very good	3	7.0
Good	9	20.9
Moderate	17	39.5
Poor	13	30.2
Very poor	1	2.3
Satisfaction with health status	n	%
Very satisfied	0	0.0
Satisfied	8	18.6
Neither satisfied nor dissatisfied	16	37.2
Dissatisfied	13	30.2
Very dissatisfied	6	14.0

The mean scores for the WHOQOL-BREF domains were as follows: 50.8 (SD = 21.4) for physical health, 49.1 (SD = 13.5) for psychological health, 57.3 (SD = 22.8) for social relationships, and 57.2 (SD = 14.6) for the environment (Table 5).

**Table 5:** WHOQOL-BREF domain scores for quality of life assessment in women with lipedema.

Domain	Mean Score	SD	Min	Max
Physical health	50.8	16.9	3.6	89.3
Psychological health	46.3	17.5	23.1	76.9
Social relationships	50.4	20.8	0	100
Environment	49.6	14.2	25.0	84.4

SD, standard deviation.

### Relationships between symptoms and depression

**Figure 4:**
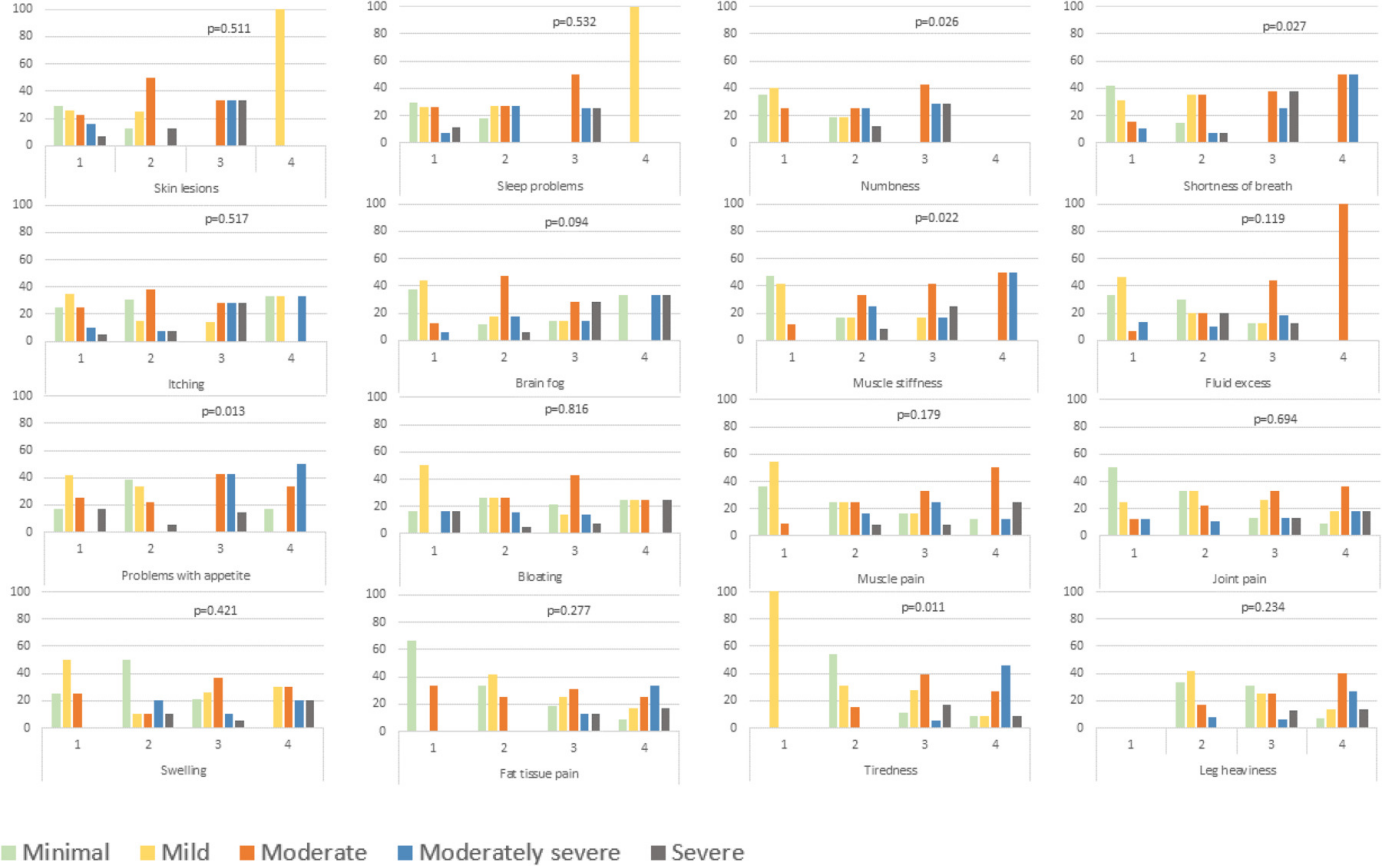
PHQ-9 scores according to different severity of lipedema symptoms. Chi-square tests were used to assess whether symptom prevalence significantly differed across depressive symptoms severity levels.

The presence of 16 specific physical symptoms was evaluated using a 5-point Likert scale, in which 0 signifies no problem and 4 denotes extreme severity. These symptoms were subsequently analyzed across the PHQ-9 scores (minimal to severe depressive symptoms). Participants with moderate shortness of breath, muscle stiffness, and appetite issues had significantly higher rates of moderate to severe depressive symptoms (*p* = 0.027, *p* = 0.022 and *p* = 0.013, respectively). Similarly, tiredness and numbness were associated with elevated depressive symptoms levels, highlighting the intricate relationship between physical and mental health in this population (*p* = 0.011 and *p* = 0.026, respectively) (Figure 4).

## Discussion

This study underscores the considerable mental health burden faced by women with lipedema in Czechia, with over half of participants exhibiting mild to moderate depressive symptoms, and 9.3% classified with severe depressive symptoms. These findings align with prior research from countries such as Poland (14), reinforcing the significant psychological toll of lipedema and emphasizing a pressing need for comprehensive care that addresses both physical and mental health.

The study's findings reveal specific lipedema symptoms—such as shortness of breath, muscle stiffness, and appetite changes—that are significantly associated with depressive symptoms severity. This connection suggests that the physical limitations caused by lipedema may contribute directly to psychological distress, potentially leading to social isolation and reduced self-esteem (10, 26, 27). Given that high levels of pain, tiredness, and swelling were frequently reported, addressing these physical symptoms through pain management and lifestyle interventions could be a viable pathway to alleviating depressive symptoms in this population (15, 28, 29).

Despite the considerable health challenges, a majority of participants reported engaging in regular physical activity, primarily walking. This finding is promising, as physical activity has been shown to benefit mental health, potentially reducing depressive symptoms and enhancing well-being (30, 31). Regular physical activity can also contribute to symptom self-management and overall health improvement, suggesting that promoting accessible, low-impact exercise options may serve as an effective strategy for managing both physical and mental symptoms in lipedema (32, 33).

Additionally, the prevalence of comorbid conditions, such as venous insufficiency and lymphedema, further complicates lipedema management. The significant overlap of these conditions points to the necessity for an integrated healthcare approach that considers comorbidities, enhancing patient outcomes by providing holistic, multi-disciplinary care (11, 14, 34). The WHOQOL-BREF scores indicated notable impairment in the psychological health of women with lipedema, highlighting a significant emotional burden. This burden may be attributed to chronic physical symptoms, limited mobility, and dissatisfaction with body image. Existing research shows that pain, fatigue, and social misunderstandings can further exacerbate psychological distress and diminish overall quality of life for these individuals (14, 26).

The results highlight the importance of incorporating mental health considerations into lipedema care. Providing patients with access to mental health resources, alongside traditional treatments, may address both the psychological and physical challenges of lipedema (15, 27). This aligns with findings from previous studies that emphasize the value of dual-focus care strategies, supporting both physical and mental health in chronic conditions (14, 15, 26, 28).

Several limitations should be considered when interpreting the findings of this study. Firstly, the sample was recruited through online platforms, potentially leading to sampling bias and limiting the generalizability of results. Additionally, the sample size was relatively small, with only 43 participants, making it difficult to draw generalized conclusions. Reliance on self-reported data may introduce recall and social desirability biases, and the lack of clinical assessment for lipedema diagnosis may have resulted in misclassification of participants. However, this approach has been widely adopted in studies involving underdiagnosed and hard-to-reach populations (14). Furthermore, certain demographic groups may be underrepresented, impacting the diversity of the sample. It is also important to note that the study is correlational, so we cannot determine a causal relationship (35) between lipedema and depression as PHQ-9 is a screening tool; these results reflect symptom burden rather than confirmed clinical diagnoses of depression; thus, we cannot conclude whether lipedema causes depression or vice versa. Finally, measurement limitations and the absence of controls for potential confounding factors further constrain the findings. Addressing these limitations in future research endeavors is essential to advance the understanding and management of lipedema effectively.

## Conclusions

This study provides critical insights into the relationship between physical symptoms, depressive symptoms, and quality of life in women with lipedema in Czechia. The high prevalence of depressive symptoms, coupled with low quality of life ratings, underscores the significant mental and physical health challenges these individuals face. The associations between specific symptoms and depressive symptoms severity emphasize the need for integrated care approaches that prioritize both physical and psychological well-being.

Future research should focus on larger, more diverse samples to improve the generalizability of these findings and explore causal pathways between physical and mental health symptoms in lipedema. Such studies could help inform the development of holistic treatment protocols that include both mental health support and targeted physical interventions, addressing the unique needs of individuals with lipedema more effectively.

## Data Availability

The raw data supporting the conclusions of this article will be made available by the authors, without undue reservation.
